# Improving attendance for cardiovascular risk assessment in Australian general practice: an RCT of a monetary incentive for patients

**DOI:** 10.1186/1471-2296-13-54

**Published:** 2012-06-08

**Authors:** Nigel Stocks, James Allan, Oliver Frank, Sue Williams, Philip Ryan

**Affiliations:** 1 Discipline of General Practice, The University of Adelaide, Adelaide, SA, 5005, Australia; 2 Hills Medical Service, Aldgate, 5054, Australia; 3 Discipline of Public Health, The University of Adelaide, Adelaide, SA, 5005, Australia

## Abstract

**Background:**

Preventive health care is an important part of general practice however uptake of activities by patients is variable. Monetary incentives for doctors have been used in the UK and Australia to improve rates of screening and immunisation. Few studies have focussed on incentives for patients to attend preventive health care examinations. Our objective was to investigate the use of a monetary incentive to increase patient attendance with their general practitioner for a cardiovascular risk assessment (CVRA).

**Methods:**

A pragmatic RCT was conducted in two Australian general practices. Participating GPs underwent academic detailing for cardiovascular risk assessment. 301 patients aged 40–74, who did not have cardiovascular disease, were independently randomised to receive a letter inviting them to a no cost cardiovascular risk assessment with their GP, or the same letter plus an offer of a $25 shopping voucher if they attended. An audit of patient medical records was also undertaken and a patient questionnaire administered to a sub sample of participants. Our main outcome measure was attendance for cardiovascular risk assessment.

**Results:**

In the RCT, 56/301(18.6%) patients attended for cardiovascular risk assessment, 29/182 (15.9%) in the control group and 27/119 (22.7%) in the intervention group. The estimated difference of 6.8% (95% CI: -2.5% to 16.0%) was not statistically significant, P = 0.15. The audit showed that GPs may underestimate patients’ absolute cardiovascular risk and the questionnaire that mailed invitations from GPs for a CVRA may encourage patients to attend.

**Conclusions:**

A small monetary incentive does not improve attendance for cardiovascular risk assessment. Further research should be undertaken to determine if there are other incentives that may increase attendance for preventive activities in the general practice setting.

**Clinical trials registration:**

ACTRN12608000183381

## Backgroud

Most Primary Health Care (PHC) interventions to improve the primary and secondary prevention of cardiovascular disease (CVD) have focussed on improvements in identifying patients at risk [1] and multi-factorial interventions, usually incorporating nurse led clinics for secondary prevention [2]. Whilst GPs in Australia and the UK have been offered financial incentives to reach quality improvement targets, little research has explored what incentives might encourage consumers to attend GPs for preventive health care. Some research has examined incentives to improve preventive behaviour, for instance to increase rates of immunisation, cancer screening and smoking cessation [3,4]. Only one general practice study conducted in Denmark showed that attendance at preventive health examinations for CVD was higher when the examination was free [5]. There have been no Australian or UK studies exploring the use of incentives to promote preventive health examinations.

Our study aimed to trial the use of a monetary incentive to increase the uptake of a cardiovascular risk assessment (CVRA) in patients without a history of CVD.

## Methods

### Study type and setting

A randomised controlled trial of a monetary incentive to improve attendance for CVRA was conducted in two Adelaide urban general practices (A and B). Both practices had similar demographic and socioeconomic characteristics (Index of socio-economic advantage-disadvantage for the top 10 postcodes of patients in each practice 984 and 985 respectively).

### Participants

Practice databases were searched to identify patients aged 40–74 years without diagnosed CVD. GPs reviewed the generated patient lists to exclude those that they considered unsuitable because of serious illness, language barriers, or recent bereavement that might affect their participation in the study. The list was further limited to include only one patient per household, by alternately selecting a male or female patient from a shared address.

### Intervention

Letters were sent to a total of 301 patients inviting them to attend for CVRA with their usual GP. Intervention arm patients (n = 119) were offered a shopping voucher (to the value of $25) for attendance within 6 months of the invitation date. Along with the invitation, all patients received an information sheet, consent form and a brochure outlining what a CVRA entailed. Attendance slips were also provided, to be taken to the assessment visit to identify participants as part of the trial, and to act as evidence of attendance. These slips were collected during regular visits to the practices. Patients had 6 months in which to attend for CVRA after the invitation was sent. Reminders were not sent. All GPs were trained in the use of a paper version of the NZ absolute cardiovascular risk tables [6].

### Outcome

Attendance at the general practice for a CVRA within 6 months of the invitation, as verified by collection of an attendance slip by research staff during a 9 month follow up period.

### Randomisation

Within each practice eligible patients were randomised to control or intervention arms using a random number table by an independent statistician. Assignment was weighted to the control arm (3:2) because a lower participation rate was expected in this arm.

### Sample size calculation

We assumed that 65% of patients would respond to a letter asking them to attend for CVRA without any incentive. To be able to detect an increase in the response rate above 80% (a 15% + absolute increase) in those receiving an incentive, with 95% confidence and 80% power we calculated that we would need 150 patients from each practice (300 in total).

### Audit and questionnaire

An audit of consenting participants’ medical records was conducted between 3 and 9 months after their attendance for the CV risk assessment. Details collected included attendance date, gender, age, family history of CVD, diabetes, smoking status, height, weight, blood pressure, lipid analysis, current medications and relevant referrals. The audit also recorded any CV risk calculation made at the risk assessment visit, or changes to medications at, or since, the visit. Dates of the most recent previous consultation, and any subsequent consultations were also recorded.

A follow-up questionnaire was sent to a subgroup of participants (practice B) to ask why they participated in the trial (not reported here), provide an estimate of their own CVD risk, the perceived benefit of CVRA and whether they would have seen their GP for a CVRA ‘heart health check’ if they had not been invited.

### Analysis

Descriptive statistics were used to report most data. A binomial generalised linear model with an identity link was used to estimate the difference in the proportions of patients attending between the intervention and control groups. The standard error of the estimate was not adjusted for clustering at the practice level, as we had only two practices and no suitable method exists to adjust in these circumstances. It is possible therefore that our test may be anti-conservative.

### Ethics

This study received ethics approval from the University of Adelaide Human Research Ethics Committee. Informed consent was obtained from all participants and patients only entered into the trial if we received a signed consent form by a reply paid envelop. Some patients consented to be in the trial but did not consent to the medical record audit.

## Results

**Figure 1:**
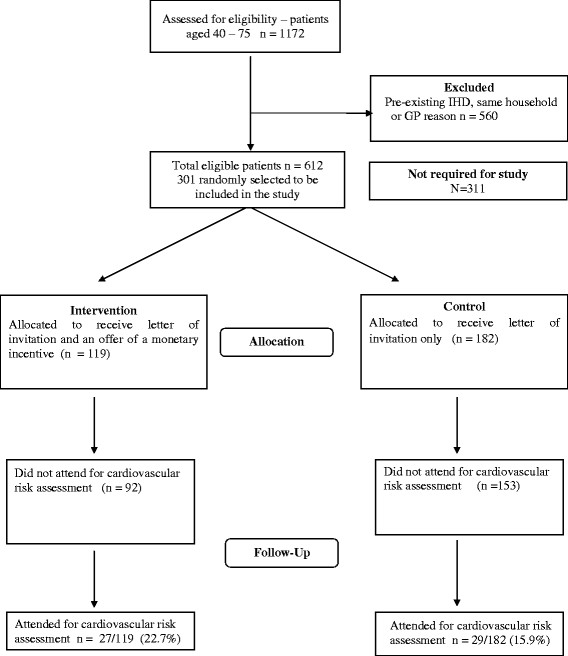
Study flow chart.

Attendance frequencies, as determined from collection of the attendance slips, are shown in Table 1. Fifty nine percent of all participants were female; and 57% were aged over 60 years. A CONSORT flow chart appears in Figure 1.

**Table 1:** Attendance for cardiovascular risk assessment

	**Combined**
Total no Attendees/Invited	56/301 (18.6%)
Control Arm Attended/Invited	29/182 (15.9%)
Intervention Arm attended/Invited	27/119 (22.7%)

The estimated absolute difference of 6.8% (95% CI: -2.5% to 16.0%) was not statistically significant, P = 0.15 using a binominal generalised linear model.

There were no adverse events or side effects in either the intervention or control group.

### Characteristics of attendees

The age and gender of participants attending for CVRA is summarised in Table 2. The data presented indicates a similar age and gender distribution by practice and intervention group, although there were slightly fewer control group participants in the 40–49 age band and slightly more in the 70–74 age group.

**Table 2:** Characteristics of attendees for CVD risk assessment

	**Practice A (I:C)**	**Practice B (I:C)**	**Combined (I:C)**
Gender			
Female	14 (6:8) 58%	19 (10:9) 59%	33 (16:17) 59%
Male	10 (6:4) 42%	13 (5:8) 41%	23 (11:12) 41%
Totals	24 100%	32 100%	56 100%
Age (years)			
40–49	5 (4:1) 21%	5 (3:2) 16%	10 (7:3) 18%
50–59	7 (3:4) 29%	7 (4:3) 21%	14 (7:7) 25%
60–69	7 (4:3) 29%	9 (3:6) 27%	16 (7:9) 29%
70-74	5 (1:4) 21%	11 (5:6) 33%	16 (6:10) 29%
Totals	24 100%	32 100%	56 100%

### Audit

**Figure 2:**
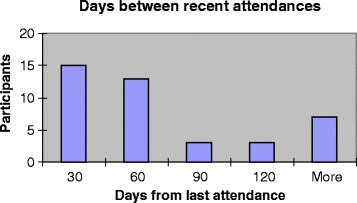
Participants’ recent attendance frequency.

The audit of 41 (consenting) participants medical records (out of 56 participants) enabled us to determine the time between their most recent previous consultation and their attendance for CVRA, as an indicator of their frequency of attendance. Most of these participants (68%) had attended the practice within 60 days prior to their attendance for CVRA (Figure 2), suggesting that the majority of our participants were regularly attending patients. Confirmation of attendance dates also indicated that 85% of participants attended within 2 months of invitation and all within 6 months. Time to attendance was similar for control and intervention groups.

### Comparison of GP and audit assessed risk

**Figure 3:**
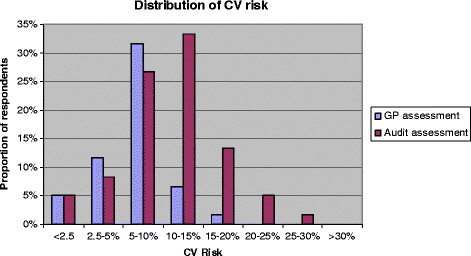
Comparison of GP and audit estimates of participants' absolute CVD risk score.

Using data collected by an audit of the GP records of 41 participants we compared the GPs’ risk score recorded during the CVRA visit (available for only 34 attendees) with one that we calculated from the available data in the medical records. Figure 3 shows a trend toward lower absolute CVD risk estimates by the GPs.

### Questionnaire

Questionnaires were sent to 32 participants from practice B and we received 24 responses (75%) Participants were asked to indicate their level of risk (from extremely low risk to extremely high risk) by marking a cross on a visual analogue scale. Their responses were compared, for intervention (I) and control (C) groups and summarised in Table 3 below. They were also asked if they would have had the CVD check up for their ‘heart health’ if they had not received an invitation to visit their GP, 12 of 23 respondents said no.

**Table 3:** Participants rating of their own CVD risk in the next 5 years

	**Intervention**	%	**Control**	%
% 5 year CVD risk on visual analogue scale				
0-24%	7	27%	5	18%
25-49%	10	38%	17	61%
50-74%	8	31%	5	18%
75-100%	1	4%	1	4%
Total	26	100%	28	100%

The *X*^2^ test for trend (df3) was 2.77 p = 0.428.

## Discussion

### Main results

The results of our RCT showed that attendance after a mailed invitation to patients aged 40–75 for CVRA was low (15.9%) and that a $25 monetary incentive did not lead to a statistically significantly increase in attendance, although there was an absolute difference in attendance between the two groups of 6.8%. We also found that GPs in this study tended to underestimate their patients CVD risk based on available information in the medical records, that participants in the intervention and control groups had similar perceptions of their future CVD risk and that almost half the participants from one practice would not have had a CVRA without the prompt provided by the invitation letter.

Our desired behaviour was attendance for CVRA, which has been promoted recently [7]. Although the cost-effectiveness of CVRA is yet to be determined CVRA can facilitate the management of individual patients [8]. We could have chosen the 45–49 year old health check, a separate item number in the Australian fee for service system ($104.00 AUS), or any other preventive health activity, but the CVRA was applicable to everyone in the selected age range, except those with established CVD. We were interested in a proof of principle and although responses may vary between desired activities the addition of an appropriate incentive might be expected to improve response rates.

We can only speculate why the uptake for CVRA was low and why a small monetary incentive did not significantly improve attendance rates. In Australia up to 85% of patients visit their GP at least once during a calendar year with most Australians visiting 2-3 times per year [9,10]. This means there are potentially many opportunities for GPs to assess cardiovascular risk factors and calculate a cardiovascular risk score. It is possible that the patients we approached had already undertaken a CVRA or at least had their BP, fasting glucose and cholesterol measured. Patients would therefore have little motivation for a repeat assessment. However we know that patients receive only about 60% of indicated preventive services that are indicated for them [11-14] and a recent study in Australia has shown that there are gaps in recording and managing BP and lipids in Australian general practice [15]. There is also evidence that patients who are at high risk of a cardiovascular event are undertreated [16]. Another explanation may in involve patients’ perception of their risk for cardiovascular disease. In a study from the Netherlands many patients were deemed to be at low risk and there was a mismatch between perceived and actual risk with 4 in 5 high risk patients believing incorrectly that were at low risk and 1 in 5 low risk patients believing they were at high risk [17]. This could reduce the number of patients believing that CVRA would be beneficial to them and hence affect attendance rates.

We found that GPs in this study tended to underestimate the CVD risk of their patients. This could be explained if GPs failed to incorporate modifying factors such family history of premature heart disease, impaired glucose tolerance or BMI > 30 patients' absolute risk. Other studies have demonstrated under and over estimations of CVD risk [18-20]. This may have consequences for the preventive care offered and potentially health outcomes so we should consider better training and/or the use of electronic calculators to improve CVD risk estimation. Our questionnaire also highlighted that inviting patients for CVRA may be worthwhile with almost half indicating that the invitation alone encouraged them to attend and have their ‘heart health’ assessed.

### Limitations

This study had several limitations. The absolute number of patients responding in the RCT was small although the observed difference in attendance was almost 7%. Non-attendees may have already had their cardiovascular risk assessed, or may have been concerned that it was a research study. Response rates to a letter(s) or telephone reminder for preventive health care in general practice can vary considerably; for women who had not had a cervical smear in the past 3 years only 10.7% responded to a letter over 6 months vs 6.3% in the control [9] whereas for a group of preventive activities the general procedure completion rate was 42% in a study from Ottawa. [10] Resource limitations limited our RCT to two practices and prevented sending reminders which have been shown to increase response rates [21]. Invitation letters have been shown to be better than opportunistic health checks in general practice [22] although a Canadian study demonstrated that a telephone invitation to a health awareness program may be better than a letter, but the response to a letter at 44% was still substantial [23]. The findings from our questionnaire confirm these previous studies.

Finally we could not conduct a double blinded trial because of the obvious difficulties, participants had to be informed about the study and those in incentive arm had to know they would receive a payment if they attended. We did not inform GPs which participants were in the incentive arm, but we could not stop patients discussing this with their doctor. Attendance slips were collected by research staff who did not know which group a patient was in and initial analysis was blinded.

## Conclusions

We believe that this is the first study of its kind in Australia and provides evidence that the use of a small monetary incentive to promote a preventive activity does not work. It is likely that incentives for medication adherence for diagnosed conditions - where the benefits of treatment may be more immediately evident and the debate about payments more topical [24,25] - differ from the offer of an incentive for preventive care, where individual benefits are less clear but gains for the community potentially large. Despite this negative result further research with patients is required to determine what, if any, incentives may encourage attendance for preventive activities in primary care.

## Competing interests

The authors have no competing interests

## Authors’ contributions

NS conceived the idea for the study. NS, JA, SW and PR devised the methods and study protocol. JA, SW, OF implemented the study with oversight from NS. NS, JA, SW and PR helped analyse and interpret the results. All authors contributed to the writing and revision of the paper. All authors’ read and approved the final manuscript.

## Funding

This project was supported by a Royal Australian College of General Practitioners Cardiovascular Research Grant.

## Pre-publication history

The pre-publication history for this paper can be accessed here:

http://www.biomedcentral.com/1471-2296/13/54/prepub
